# 3-(2-Fluoro­phenyl­sulfon­yl)-2,5,7-trimethyl-1-benzo­furan

**DOI:** 10.1107/S1600536814007909

**Published:** 2014-04-16

**Authors:** Hong Dae Choi, Pil Ja Seo, Uk Lee

**Affiliations:** a Department of Chemistry, Dongeui University, San 24 Kaya-dong, Busanjin-gu, Busan 614-714, Republic of Korea; b Department of Chemistry, Pukyong National University, 599-1 Daeyeon 3-dong, Nam-gu, Busan 608-737, Republic of Korea

## Abstract

In the title compound, C_17_H_15_FO_3_S, the dihedral angle between the mean planes of the benzo­furan and 2-fluoro­phenyl rings is 87.61 (4) Å. In the crystal, mol­ecules are linked *via* pairs of C—H⋯π inter­actions into inversion-related dimers. These dimers are linked by C—H⋯O hydrogen bonds into supra­molecular chains running along the *a*-axis direction.

## Related literature   

For background information and the crystal structures of related compounds, see: Choi *et al.* (2010 ▶, 2012 ▶); Seo *et al.* (2011 ▶).
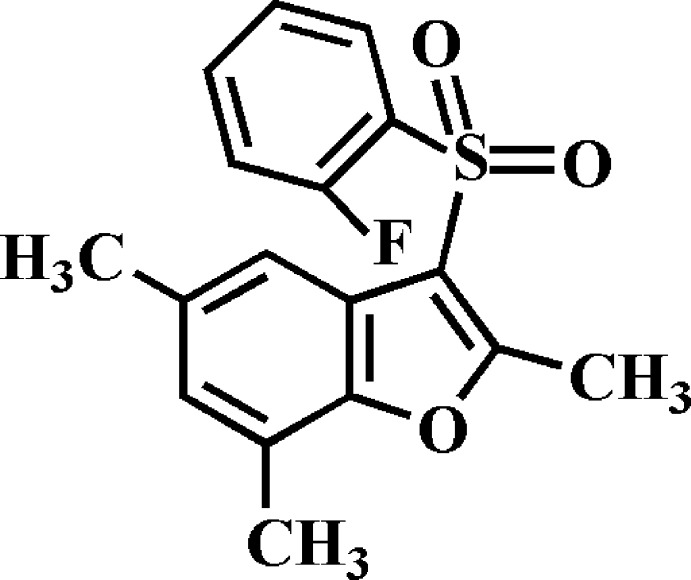


## Experimental   

### 

#### Crystal data  

C_17_H_15_FO_3_S
*M* *_r_* = 318.35
Triclinic, 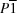

*a* = 7.7658 (2) Å
*b* = 8.3529 (3) Å
*c* = 12.9732 (4) Å
α = 74.253 (2)°
β = 75.048 (2)°
γ = 66.046 (1)°
*V* = 729.66 (4) Å^3^
*Z* = 2
Mo *K*α radiation
μ = 0.24 mm^−1^
*T* = 173 K
0.34 × 0.26 × 0.23 mm

#### Data collection  

Bruker SMART APEXII CCD diffractometer
Absorption correction: multi-scan (*SADABS*; Bruker, 2009 ▶) *T*_min_ = 0.700, *T*_max_ = 0.746
13247 measured reflections
3498 independent reflections
3057 reflections with *I* > 2σ(*I*)
*R*_int_ = 0.023

#### Refinement  

*R*[*F* ^2^ > 2σ(*F* ^2^)] = 0.038
*wR*(*F* ^2^) = 0.104
*S* = 1.04
3498 reflections
202 parameters
H-atom parameters constrained
Δρ_max_ = 0.34 e Å^−3^
Δρ_min_ = −0.36 e Å^−3^

### 

Data collection: *APEX2* (Bruker, 2009 ▶); cell refinement: *SAINT* (Bruker, 2009 ▶); data reduction: *SAINT*; program(s) used to solve structure: *SHELXS97* (Sheldrick, 2008 ▶); program(s) used to refine structure: *SHELXL97* (Sheldrick, 2008 ▶); molecular graphics: *ORTEP-3 for Windows* (Farrugia, 2012 ▶) and *DIAMOND* (Brandenburg, 1998 ▶); software used to prepare material for publication: *SHELXL97*.

## Supplementary Material

Crystal structure: contains datablock(s) I. DOI: 10.1107/S1600536814007909/zq2222sup1.cif

Structure factors: contains datablock(s) I. DOI: 10.1107/S1600536814007909/zq2222Isup2.hkl

Supporting information file. DOI: 10.1107/S1600536814007909/zq2222Isup3.cml

CCDC reference: 996303

Additional supporting information:  crystallographic information; 3D view; checkCIF report

## Figures and Tables

**Table 1:** Hydrogen-bond geometry (Å, °) *Cg*1 is the centroid of the C2–C7 benzene ring.

*D*—H⋯*A*	*D*—H	H⋯*A*	*D*⋯*A*	*D*—H⋯*A*
C16—H16⋯O2^i^	0.95	2.58	3.462 (2)	155
C9—H9*C*⋯*Cg*1^ii^	0.98	2.79	3.612 (2)	142

Symmetry codes: (i) 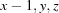; (ii) 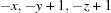.

**Fig. 1.**
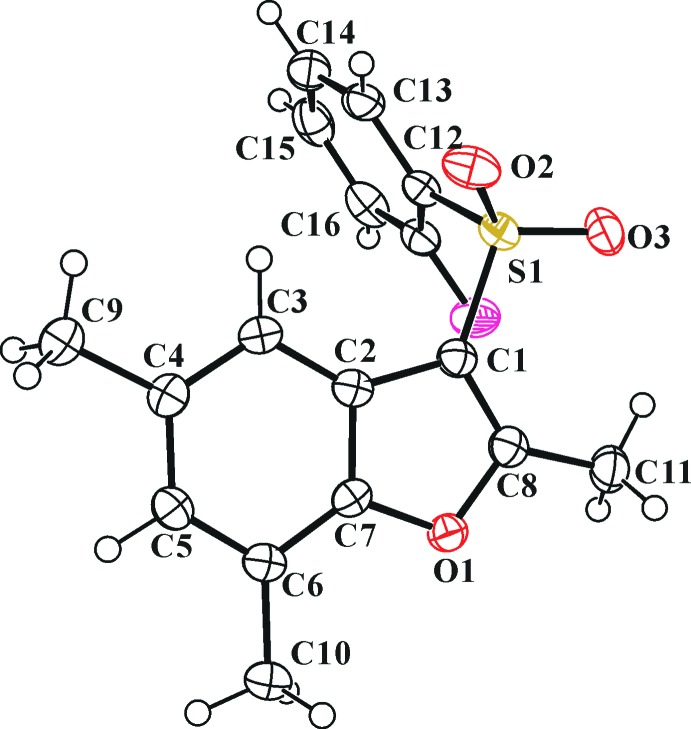
The molecular structure of the title molecule with the atom numbering scheme The displacement ellipsoids are drawn at the 50% probability level. The hydrogen atoms are presented as small spheres of arbitrary radius.

**Fig. 2.**
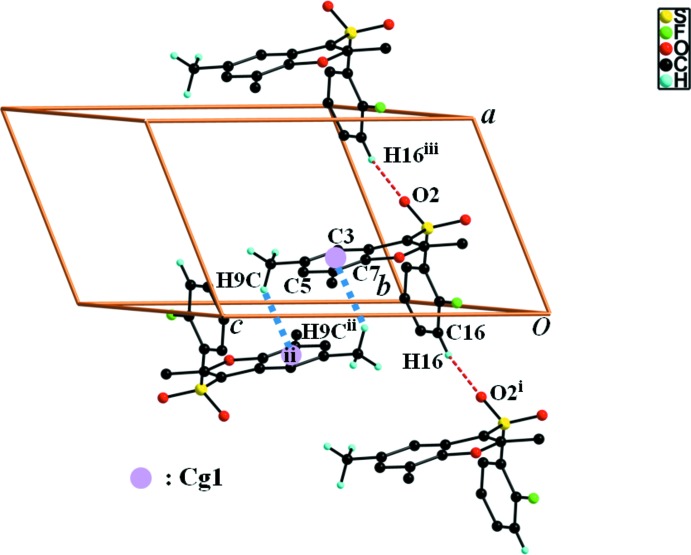
A view of the C—H···O and C—H···π interactions (dotted lines) in the crystal structure of the title compound. H atoms non-participating in hydrogen-bonding were omitted for clarity. [Symmetry codes: (i) x - 1, y, z; (ii) - x, - y + 1, - z + 1; (iii) x + 1, y, z.]

## supplementary crystallographic information

### 1. Comment

As a part of our ongoing study of 2,5,7-trimethyl-1-benzofuran derivatives containing 4-fluorophenylsulfonyl (Choi *et al.*, 2010), 3-fluorophenylsulfonyl (Seo *et al.*, 2011) and 4-methylphenylsulfonyl (Choi *et al.*, 2012) substituents in the 3-position, we report here on the crystal structure of the title compound.

In the title molecule (Fig. 1), the benzofuran ring system is essentially planar, with a mean deviation of 0.013 (1) Å from the least-squares plane defined by the nine constituent atoms. The 2-fluorophenyl ring is essentially planar, with a mean deviation of 0.004 (1) Å from the least-squares plane defined by the six constituent atoms. The dihedral angle formed by the benzofuran ring system and the 2-fluorophenyl ring is 87.61 (4)°. In the crystal structure (Fig. 2), molecules are linked via pairs of C—H···π interactions (Table 1, Cg1 is the centroid of the C2–C7 benzene ring) into inversion-related dimers. These dimers are further linked by C—H···O hydrogen bonds (Table 1), forming supramolecular chains running along the *a*-axis direction.

### 2. Experimental

3-Chloroperoxybenzoic acid (77%, 448 mg, 2.0 mmol) was added in small portions to a stirred solution of 3-(2-fluorophenylsulfanyl)-2,5,7-trimethyl-1-benzofuran (257 mg, 0.9 mmol) in dichloromethane (35 mL) at 273 K. After being stirred at room temperature for 10h, the mixture was washed with saturated sodium bicarbonate solution and the organic layer was separated, dried over magnesium sulfate, filtered and concentrated at reduced pressure. The residue was purified by column chromatography (benzene) to afford the title compound as a colorless solid [yield 71%, m.p. 409–410 K; *R*_f_ = 0.52 (benzene)]. Single crystals suitable for X-ray diffraction were prepared by slow vaporation of a solution of the title compound in acetone at room temperature.

### 3. Refinement

All H atoms were positioned geometrically and refined using a riding model, with C—H = 0.95 Å for aryl and 0.98 Å for methyl H atoms, respectively. *U*_iso_ (H) = 1.2*U*_eq_ (C) for aryl and 1.5*U*_eq_ (C) for methyl H atoms. The positions of methyl hydrogens were optimized using the SHELXL-97's command AFIX 137 (Sheldrick, 2008).

### Crystal data

**Table:**

C_17_H_15_FO_3_S	*Z* = 2
*M**_r_* = 318.35	*F*(000) = 332
Triclinic, *P*1	*D*_x_ = 1.449 Mg m^−^^3^
Hall symbol: -P 1	Melting point = 410–409 K
*a* = 7.7658 (2) Å	Mo *K*α radiation, λ = 0.71073 Å
*b* = 8.3529 (3) Å	Cell parameters from 6186 reflections
*c* = 12.9732 (4) Å	θ = 2.7–28.2°
α = 74.253 (2)°	µ = 0.24 mm^−^^1^
β = 75.048 (2)°	*T* = 173 K
γ = 66.046 (1)°	Block, colourless
*V* = 729.66 (4) Å^3^	0.34 × 0.26 × 0.23 mm

### Data collection

**Table:**

Bruker SMART APEXII CCD diffractometer	3498 independent reflections
Radiation source: rotating anode	3057 reflections with *I* > 2σ(*I*)
Graphite multilayer monochromator	*R*_int_ = 0.023
Detector resolution: 10.0 pixels mm^-1^	θ_max_ = 28.0°, θ_min_ = 1.7°
φ and ω scans	*h* = −10→10
Absorption correction: multi-scan (*SADABS*; Bruker, 2009)	*k* = −11→10
*T*_min_ = 0.700, *T*_max_ = 0.746	*l* = −16→17
13247 measured reflections	

### Refinement

**Table:**

Refinement on *F*^2^	Primary atom site location: structure-invariant direct methods
Least-squares matrix: full	Secondary atom site location: difference Fourier map
*R*[*F*^2^ > 2σ(*F*^2^)] = 0.038	Hydrogen site location: difference Fourier map
*wR*(*F*^2^) = 0.104	H-atom parameters constrained
*S* = 1.04	*w* = 1/[σ^2^(*F*_o_^2^) + (0.0555*P*)^2^ + 0.2699*P*] where *P* = (*F*_o_^2^ + 2*F*_c_^2^)/3
3498 reflections	(Δ/σ)_max_ < 0.001
202 parameters	Δρ_max_ = 0.34 e Å^−^^3^
0 restraints	Δρ_min_ = −0.36 e Å^−^^3^

### Special details

**Table:**

Geometry. All esds (except the esd in the dihedral angle between two l.s. planes) are estimated using the full covariance matrix. The cell esds are taken into account individually in the estimation of esds in distances, angles and torsion angles; correlations between esds in cell parameters are only used when they are defined by crystal symmetry. An approximate (isotropic) treatment of cell esds is used for estimating esds involving l.s. planes.
Refinement. Refinement of F^2^ against ALL reflections. The weighted R-factor wR and goodness of fit S are based on F^2^, conventional R-factors R are based on F, with F set to zero for negative F^2^. The threshold expression of F^2^ > 2sigma(F^2^) is used only for calculating R-factors(gt) etc. and is not relevant to the choice of reflections for refinement. R-factors based on F^2^ are statistically about twice as large as those based on F, and R- factors based on ALL data will be even larger.

### Fractional atomic coordinates and isotropic or equivalent isotropic displacement parameters (Å^2^)

**Table:**

	*x*	*y*	*z*	*U*_iso_*/*U*_eq_	
S1	0.42141 (5)	0.28123 (5)	0.15133 (3)	0.02646 (12)	
F1	0.04064 (14)	0.26625 (13)	0.15747 (8)	0.0372 (2)	
O1	0.29698 (15)	−0.04837 (13)	0.41192 (8)	0.0261 (2)	
O2	0.55334 (16)	0.35762 (16)	0.15685 (9)	0.0370 (3)	
O3	0.47053 (17)	0.17497 (16)	0.07064 (9)	0.0368 (3)	
C12	0.2034 (2)	0.4608 (2)	0.13354 (11)	0.0248 (3)	
C17	0.0368 (2)	0.4340 (2)	0.13746 (12)	0.0281 (3)	
C1	0.3680 (2)	0.16080 (19)	0.27862 (12)	0.0248 (3)	
C2	0.3203 (2)	0.22449 (19)	0.37943 (11)	0.0236 (3)	
C3	0.3066 (2)	0.37738 (19)	0.40990 (12)	0.0261 (3)	
H3	0.3364	0.4706	0.3573	0.031*	
C4	0.2485 (2)	0.3905 (2)	0.51883 (12)	0.0273 (3)	
C5	0.2053 (2)	0.2505 (2)	0.59508 (12)	0.0274 (3)	
H5	0.1650	0.2625	0.6691	0.033*	
C6	0.2185 (2)	0.09579 (19)	0.56803 (12)	0.0249 (3)	
C7	0.2774 (2)	0.08988 (18)	0.45848 (12)	0.0232 (3)	
C8	0.3499 (2)	−0.0012 (2)	0.30262 (12)	0.0264 (3)	
C9	0.2271 (3)	0.5540 (2)	0.55619 (14)	0.0358 (4)	
H9A	0.2797	0.6306	0.4961	0.054*	
H9B	0.2964	0.5184	0.6169	0.054*	
H9C	0.0914	0.6196	0.5799	0.054*	
C10	0.1698 (2)	−0.0525 (2)	0.64939 (12)	0.0301 (3)	
H10A	0.0649	−0.0689	0.6300	0.045*	
H10B	0.1306	−0.0223	0.7219	0.045*	
H10C	0.2821	−0.1632	0.6493	0.045*	
C11	0.3704 (2)	−0.1314 (2)	0.23854 (14)	0.0351 (4)	
H11A	0.3914	−0.0806	0.1609	0.053*	
H11B	0.2538	−0.1592	0.2568	0.053*	
H11C	0.4796	−0.2408	0.2554	0.053*	
C13	0.1995 (2)	0.6340 (2)	0.11204 (12)	0.0310 (3)	
H13	0.3121	0.6552	0.1092	0.037*	
C14	0.0312 (3)	0.7759 (2)	0.09474 (13)	0.0387 (4)	
H14	0.0282	0.8946	0.0806	0.046*	
C15	−0.1330 (3)	0.7457 (2)	0.09789 (13)	0.0403 (4)	
H15	−0.2473	0.8439	0.0845	0.048*	
C16	−0.1318 (2)	0.5737 (2)	0.12045 (13)	0.0359 (4)	
H16	−0.2447	0.5525	0.1241	0.043*	

### Atomic displacement parameters (Å^2^)

**Table:**

	*U*^11^	*U*^22^	*U*^33^	*U*^12^	*U*^13^	*U*^23^
S1	0.0237 (2)	0.0311 (2)	0.02292 (18)	−0.01123 (15)	−0.00111 (13)	−0.00301 (14)
F1	0.0387 (5)	0.0397 (5)	0.0406 (5)	−0.0238 (4)	−0.0086 (4)	−0.0022 (4)
O1	0.0260 (5)	0.0235 (5)	0.0277 (5)	−0.0094 (4)	−0.0034 (4)	−0.0036 (4)
O2	0.0288 (6)	0.0478 (7)	0.0361 (6)	−0.0216 (5)	−0.0062 (5)	0.0025 (5)
O3	0.0379 (7)	0.0392 (6)	0.0272 (6)	−0.0096 (5)	0.0022 (5)	−0.0111 (5)
C12	0.0262 (7)	0.0287 (7)	0.0183 (6)	−0.0106 (6)	−0.0031 (5)	−0.0023 (5)
C17	0.0305 (8)	0.0335 (8)	0.0214 (6)	−0.0147 (6)	−0.0026 (6)	−0.0037 (6)
C1	0.0227 (7)	0.0255 (7)	0.0249 (7)	−0.0085 (6)	−0.0036 (5)	−0.0035 (5)
C2	0.0198 (7)	0.0255 (7)	0.0242 (7)	−0.0079 (5)	−0.0045 (5)	−0.0021 (5)
C3	0.0267 (7)	0.0238 (7)	0.0277 (7)	−0.0107 (6)	−0.0059 (6)	−0.0010 (6)
C4	0.0257 (7)	0.0267 (7)	0.0304 (7)	−0.0090 (6)	−0.0059 (6)	−0.0066 (6)
C5	0.0257 (7)	0.0320 (8)	0.0242 (7)	−0.0099 (6)	−0.0043 (6)	−0.0055 (6)
C6	0.0198 (7)	0.0267 (7)	0.0257 (7)	−0.0079 (6)	−0.0045 (5)	−0.0010 (6)
C7	0.0205 (7)	0.0211 (6)	0.0281 (7)	−0.0070 (5)	−0.0060 (5)	−0.0035 (5)
C8	0.0218 (7)	0.0273 (7)	0.0281 (7)	−0.0070 (6)	−0.0039 (6)	−0.0053 (6)
C9	0.0420 (10)	0.0316 (8)	0.0375 (9)	−0.0158 (7)	−0.0056 (7)	−0.0095 (7)
C10	0.0307 (8)	0.0308 (8)	0.0276 (7)	−0.0141 (6)	−0.0041 (6)	0.0003 (6)
C11	0.0369 (9)	0.0337 (8)	0.0369 (9)	−0.0136 (7)	−0.0005 (7)	−0.0141 (7)
C13	0.0393 (9)	0.0323 (8)	0.0228 (7)	−0.0171 (7)	−0.0049 (6)	−0.0015 (6)
C14	0.0528 (11)	0.0294 (8)	0.0276 (8)	−0.0112 (8)	−0.0075 (7)	−0.0007 (6)
C15	0.0381 (9)	0.0427 (10)	0.0255 (8)	−0.0001 (8)	−0.0070 (7)	−0.0046 (7)
C16	0.0266 (8)	0.0508 (10)	0.0261 (7)	−0.0107 (7)	−0.0037 (6)	−0.0064 (7)

### Geometric parameters (Å, º)

**Table:**

S1—O3	1.4327 (12)	C6—C7	1.384 (2)
S1—O2	1.4336 (11)	C6—C10	1.5023 (19)
S1—C1	1.7305 (15)	C8—C11	1.477 (2)
S1—C12	1.7664 (15)	C9—H9A	0.9800
F1—C17	1.3430 (18)	C9—H9B	0.9800
O1—C8	1.3646 (18)	C9—H9C	0.9800
O1—C7	1.3820 (17)	C10—H10A	0.9800
C12—C13	1.386 (2)	C10—H10B	0.9800
C12—C17	1.387 (2)	C10—H10C	0.9800
C17—C16	1.376 (2)	C11—H11A	0.9800
C1—C8	1.361 (2)	C11—H11B	0.9800
C1—C2	1.449 (2)	C11—H11C	0.9800
C2—C7	1.3887 (19)	C13—C14	1.383 (2)
C2—C3	1.393 (2)	C13—H13	0.9500
C3—C4	1.388 (2)	C14—C15	1.386 (3)
C3—H3	0.9500	C14—H14	0.9500
C4—C5	1.406 (2)	C15—C16	1.383 (3)
C4—C9	1.504 (2)	C15—H15	0.9500
C5—C6	1.387 (2)	C16—H16	0.9500
C5—H5	0.9500		
			
O3—S1—O2	118.79 (7)	C1—C8—O1	110.21 (13)
O3—S1—C1	109.75 (7)	C1—C8—C11	135.02 (14)
O2—S1—C1	108.30 (7)	O1—C8—C11	114.75 (13)
O3—S1—C12	109.12 (7)	C4—C9—H9A	109.5
O2—S1—C12	106.32 (7)	C4—C9—H9B	109.5
C1—S1—C12	103.44 (7)	H9A—C9—H9B	109.5
C8—O1—C7	107.16 (11)	C4—C9—H9C	109.5
C13—C12—C17	118.81 (14)	H9A—C9—H9C	109.5
C13—C12—S1	119.15 (12)	H9B—C9—H9C	109.5
C17—C12—S1	122.02 (12)	C6—C10—H10A	109.5
F1—C17—C16	119.00 (14)	C6—C10—H10B	109.5
F1—C17—C12	118.90 (14)	H10A—C10—H10B	109.5
C16—C17—C12	122.09 (15)	C6—C10—H10C	109.5
C8—C1—C2	107.72 (13)	H10A—C10—H10C	109.5
C8—C1—S1	127.53 (12)	H10B—C10—H10C	109.5
C2—C1—S1	124.61 (11)	C8—C11—H11A	109.5
C7—C2—C3	119.30 (13)	C8—C11—H11B	109.5
C7—C2—C1	104.47 (12)	H11A—C11—H11B	109.5
C3—C2—C1	136.22 (13)	C8—C11—H11C	109.5
C4—C3—C2	118.53 (13)	H11A—C11—H11C	109.5
C4—C3—H3	120.7	H11B—C11—H11C	109.5
C2—C3—H3	120.7	C14—C13—C12	119.81 (15)
C3—C4—C5	119.61 (14)	C14—C13—H13	120.1
C3—C4—C9	120.90 (14)	C12—C13—H13	120.1
C5—C4—C9	119.47 (14)	C13—C14—C15	120.32 (16)
C6—C5—C4	123.54 (14)	C13—C14—H14	119.8
C6—C5—H5	118.2	C15—C14—H14	119.8
C4—C5—H5	118.2	C16—C15—C14	120.53 (16)
C7—C6—C5	114.35 (13)	C16—C15—H15	119.7
C7—C6—C10	122.18 (14)	C14—C15—H15	119.7
C5—C6—C10	123.46 (14)	C17—C16—C15	118.42 (16)
O1—C7—C6	124.88 (13)	C17—C16—H16	120.8
O1—C7—C2	110.44 (12)	C15—C16—H16	120.8
C6—C7—C2	124.67 (13)		
			
O3—S1—C12—C13	−119.82 (12)	C4—C5—C6—C7	−0.3 (2)
O2—S1—C12—C13	9.42 (14)	C4—C5—C6—C10	−179.14 (14)
C1—S1—C12—C13	123.39 (12)	C8—O1—C7—C6	177.56 (13)
O3—S1—C12—C17	58.37 (14)	C8—O1—C7—C2	−0.85 (15)
O2—S1—C12—C17	−172.39 (12)	C5—C6—C7—O1	−178.51 (13)
C1—S1—C12—C17	−58.41 (13)	C10—C6—C7—O1	0.4 (2)
C13—C12—C17—F1	179.51 (13)	C5—C6—C7—C2	−0.3 (2)
S1—C12—C17—F1	1.30 (19)	C10—C6—C7—C2	178.56 (13)
C13—C12—C17—C16	0.2 (2)	C3—C2—C7—O1	179.19 (12)
S1—C12—C17—C16	−178.05 (12)	C1—C2—C7—O1	0.22 (15)
O3—S1—C1—C8	−7.65 (16)	C3—C2—C7—C6	0.8 (2)
O2—S1—C1—C8	−138.77 (14)	C1—C2—C7—C6	−178.20 (13)
C12—S1—C1—C8	108.69 (14)	C2—C1—C8—O1	−1.05 (16)
O3—S1—C1—C2	177.27 (12)	S1—C1—C8—O1	−176.80 (10)
O2—S1—C1—C2	46.15 (14)	C2—C1—C8—C11	177.56 (16)
C12—S1—C1—C2	−66.39 (13)	S1—C1—C8—C11	1.8 (3)
C8—C1—C2—C7	0.50 (16)	C7—O1—C8—C1	1.17 (15)
S1—C1—C2—C7	176.40 (11)	C7—O1—C8—C11	−177.74 (12)
C8—C1—C2—C3	−178.21 (16)	C17—C12—C13—C14	−0.2 (2)
S1—C1—C2—C3	−2.3 (2)	S1—C12—C13—C14	178.09 (12)
C7—C2—C3—C4	−0.6 (2)	C12—C13—C14—C15	−0.5 (2)
C1—C2—C3—C4	177.95 (15)	C13—C14—C15—C16	1.3 (2)
C2—C3—C4—C5	0.1 (2)	F1—C17—C16—C15	−178.80 (14)
C2—C3—C4—C9	−178.71 (14)	C12—C17—C16—C15	0.6 (2)
C3—C4—C5—C6	0.4 (2)	C14—C15—C16—C17	−1.3 (2)
C9—C4—C5—C6	179.20 (14)		

### Hydrogen-bond geometry (Å, º)

Cg1 is the centroid of the C2–C7 benzene ring.

**Table:**

*D*—H···*A*	*D*—H	H···*A*	*D*···*A*	*D*—H···*A*
C16—H16···O2^i^	0.95	2.58	3.462 (2)	155
C9—H9*C*···*Cg*1^ii^	0.98	2.79	3.612 (2)	142

Symmetry codes: (i) *x*−1, *y*, *z*; (ii) −*x*, −*y*+1, −*z*+1.
